# A novel nuclear Src and p300 signaling axis controls migratory and invasive behavior in pancreatic cancer

**DOI:** 10.18632/oncotarget.6635

**Published:** 2015-12-17

**Authors:** David Paladino, Peibin Yue, Hideki Furuya, Jared Acoba, Charles J. Rosser, James Turkson

**Affiliations:** ^1^ Natural Products and Experimental Therapeutics Program, University of Hawaii Cancer Center, University of Hawaii, Manoa, Honolulu, HI 96813, USA; ^2^ Cancer Biology Program, University of Hawaii Cancer Center, University of Hawaii, Manoa, Honolulu, HI 96813, USA; ^3^ Clinical and Translational Research Program, University of Hawaii Cancer Center, University of Hawaii, Manoa, Honolulu, HI 96813, USA

**Keywords:** Src, p300, pancreatic cancer, migration, invasion

## Abstract

The presence of Src in the nuclear compartment has been previously reported, although its significance has remained largely unknown. We sought to delineate the functions of the nuclear pool of Src within the context of malignant progression. Active Src is localized within the nuclei of human pancreatic cancer cells and mouse fibroblasts over-expressing c-Src where it is associated with p300. Nuclear Src additionally promotes the tyrosine phosphorylation of p300 in pancreatic cancer Panc-1 cells. Src, together with p300, is associated with the high-mobility group AT-hook (HMGA)2 and SET and MYND domain-containing protein (SMYD)3 gene promoters and regulates their expression in a Src-dependent manner. These nuclear Src-dependent events correlate with anchorage-independent soft-agar growth and the migratory properties in both pancreatic Panc-1 cells and mouse fibroblasts over-expressing Src. Moreover, analyses of human pancreatic ductal adenocarcinoma (PDAC) tumor tissues detected the association of nuclear Src with the HMGA2 and SMYD3 gene promoters. Our findings for the first time show the critical importance of nuclear Src and p300 function in the migratory properties of pancreatic cancer cells. Further, data together identify a previously unknown role of nuclear Src in the regulation of gene expression in association with p300 within the context of cells harboring activated or over-expressing Src. This novel mechanism of nuclear Src-p300 axis in PDAC invasiveness and metastasis may provide an opportunity for developing more effective early clinical interventions for this lethal disease.

Active Src is complexed with and phosphorylates p300 in the nucleus, and the complex is bound to HMGA2 and SMYD3 genes, thereby regulating their expression to promote pancreatic tumor cell migration and invasiveness.

## INTRODUCTION

Src is a ubiquitous non-receptor tyrosine kinase that is known to function as a signaling mediator at the cell membrane. Structurally, Src contains conserved domains and motifs that are responsible for targeting the protein to a number of subcellular locations [1]. The most well-studied cellular location of Src is at the plasma membrane, where it mediates signaling cascades through a variety of substrates [2, 3], as well as regulates focal adhesion assembly and turnover [4]. In many tumor types, including PDAC, Src is often found to be overexpressed and hyperactivated, and it contributes to oncogenic signaling [5]. Given its known role in pro-migratory processes at the plasma membrane, it is unsurprising that Src activation is predominantly associated with high tumor invasion and metastasis [6].

Aside from its establishment at the plasma membrane, some of the earliest Src (vSrc) research identified a nuclear pool of the protein [7, 8]. Subsequent studies led to the discovery of a primarily nuclear, Src-interacting protein called Sam68 [9]. However, little research since has delved into the specific nuclear function of Src. A number of recent reports have suggested Src and Src-family kinases to have functional roles within the nuclear compartment [10–13]. We have also previously described a nuclear Src complex in human pancreatic cancer cells as being associated with the c-Myc promoter and influencing gene expression [14]. Following this evidence, we aimed to delineate further specific nuclear functions of Src in pancreatic cancer to broaden our understanding of the role of an important kinase in a poorly understood tumor type.

The histone acetyltransferase (HAT) p300 is a large, modular protein that serves as a coactivator in a diverse assortment of cellular processes [15]. There are numerous reported serine/threonine phosphorylation events of p300 residues, which are also known to regulate the protein's HAT activity [16, 17]. Initially, p300 was presumed to behave as a classical tumor suppressor due primarily to its binding to and inactivation by adenovirus E1A (E1A), as well as the discovery of rare truncating mutations in the gene that occur in both primary tumors and cell lines [18]. While p300 may exhibit some tumor suppressive functions under certain contexts, numerous reports to the contrary, as well as the infrequency of inactivating p300 mutations suggested that the protein is more commonly involved in oncogenic signaling [19]. The role of p300 in PDAC progression is relatively unexplored, although it has been shown to be involved in c-Myc induction [20] and gemcitabine resistance in a three-dimensional collagen environment [21].

In this study, we demonstrate the presence of activated (pY416) Src in the nuclei of mouse embryonic fibroblasts (MEFs) overexpressing Src, PDAC cell lines, and in patient tumor samples. Src physically interacts with p300 in the PDAC and MEF cells harboring active nuclear Src and induces p300 tyrosine phosphorylation. In continuance of our previous report [14], ChIP-chip studies showed Src and p300 associate with the promoters of the pro-migratory genes HMGA2 and SMYD3. Inhibition of Src and p300 decreased HMGA2 and SMYD3 expression at both the message and protein levels, suggesting that Src and p300 cooperate to regulate their expression in Panc-1 cells. The regulation by Src and p300 of HMGA2 was similarly observed in a MEF model in which Src signaling is activated, but not in normal MEFs. Inhibition of Src and/or p300 function blocks the migration of pancreatic cancer cells and MEFs overexpressing Src. These results extend our understanding of Src signaling in cancer and provide evidence that p300 cooperates with nuclear Src to promote pancreatic cancer progression and together represent an effective target for anti-invasive therapies in pancreatic cancer, a disease characterized by its high degree of invasiveness and mortality.

## RESULTS

### Active Src is present in the nuclei of Panc-1 cells and is associated with p300

**Figure 1:**
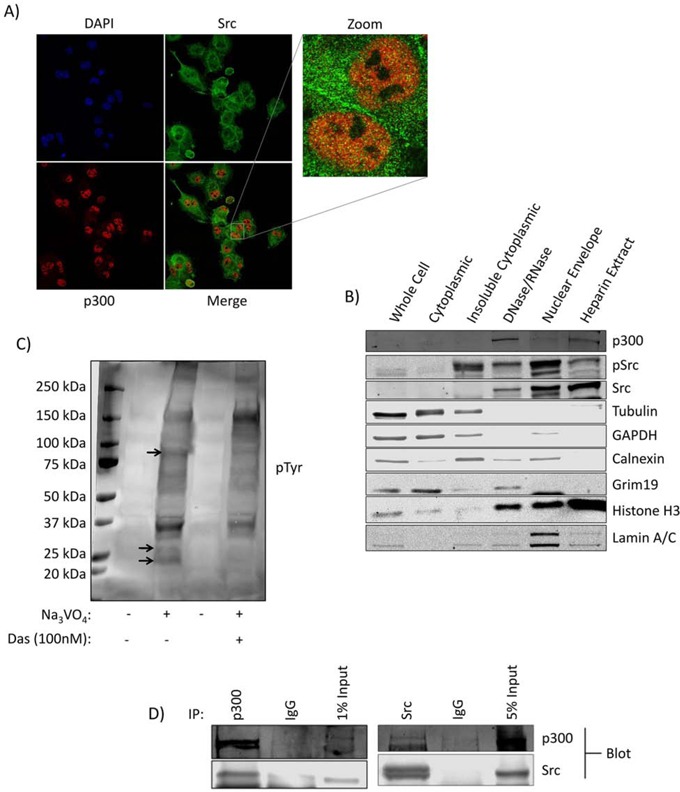
Kinase active Src is present in the nuclei of Panc-1 cells in association with p300 **A.** Immunofluorescent imaging for Src and p300 in Panc-1 cells. Cells on coverslips were fixed and stained with the indicated primary and secondary antibodies and DAPI (4′,6-diamidino-2-phenylindole), and subjected to confocal microscopy under 63X magnification. **B.** Subcellular fractionation and localization of Src and p300 in Panc-1 cells. Cell lysates of equal total protein or Panc-1 subcellular fractions were subjected to immunoblotting analysis for Src, pSrc, p300, tubulin (cytoskeletal proteins), GAPDH (soluble cytoplasmic proteins), calnexin (endoplasmic reticulum), grim19 (mitochondria), histone H3 (chromatin), and lamin A/C (nuclear lamina). **C.** Phosphotyrosine (pTyr) immunoblotting analysis using general pTyr antibody of lysates of equal total protein from the isolated Panc-1 cell nuclei following incubation in a Src kinase buffer at 37°C in the presence or absence of Na_3_VO_4_ and dasatinib as indicated. Bands absent in the dasatinib-treated lane compared to control are indicated by arrows. **D.** Src and p300 co-association in Panc-1 cells. Immunoblotting analysis of Src or p300 immunecomplexes from Panc-1 nuclear isolates probing for Src or p300, or IgG control. Positions of proteins in gel are labeled; control lanes (−) represent nuclei treated with 0.01% DMSO. Data are representative of two independent experiments.

Immunoprecipitation coupled to mass spectrometry (IP-MS) analysis of nuclear lysates from Panc-1 cells was performed to identify binding partners of nuclear Src. After comparing the specific peptides detected from the Src IP to those detected from the control IgG IP and excluding any overlapping proteins as background, we were left with a small number of Src antibody-specifically bound proteins (Table S1). From this list, the histone acetyltransferase p300 is of particular interest. To visualize Src and p300 subcellular localization, immunofluorescence with confocal microscopy was performed (Figure 1A), which detected Src throughout the cells, including within the nuclear compartment, and its co-localization with p300 (Figure 1A, Figure S1). Biochemical subfractionation was performed to further validate the nuclear presence of Src in Panc-1 cells. Once isolated, nuclei can be further subfractionated by DNAse and heparin treatment to yield various subnuclear pools [22]. Loading equal total protein in each lane, immunoblotting analysis showed a strong enrichment of total and active Src in the three nuclear subfractions (Figure 1B, DNAse/RNAse, nuclear envelope and heparin extract), as well as enrichment of p300 in the two nucleoplasmic fractions (Figure 1B, DNase/RNase, heparin extract). Active Src was specifically detected in the insoluble cytoplasmic fraction compared to the soluble fraction, as previously reported [23]. Marker proteins (tubulin, GAPDH, calnexin, grim19, histone H3, and lamin) denote their subcellular locations.

In order to determine whether nuclear Src exhibited kinase activity on endogenous substrates, samples of intact isolated Panc-1 nuclei were incubated in a Src kinase assay buffer containing sodium orthovanadate in the absence (DMSO) or presence of the Src inhibitor dasatinib and immunoprobed for general phosphotyrosine (Figure 1C). The conditions of this study preclude the possibility of accumulation of cytosolic or membranous Src substrates within the nucleus and give a profile of nuclear tyrosine phosphorylated substrates mediated by nuclear tyrosine kinases. Results showed suppression of the levels of nuclear pTyr proteins by the treatment with dasatinib (Figure 1C, arrows), indicating nuclear Src is functional and promotes the phosphorylation of native targets in the nucleus. Co-immunoprecipitation of Src and p300 from Panc-1 nuclear lysates and Western blotting analysis confirmed the interaction between the two proteins (Figure 1D). These results demonstrate that kinase-active Src is present in the nuclei of Panc-1 cells, associates with p300, and promotes tyrosine phosphorylation of target proteins.

### Nuclear localization of active Src and its association with p300 are recapitulated in mouse embryonic fibroblasts

**Figure 2:**
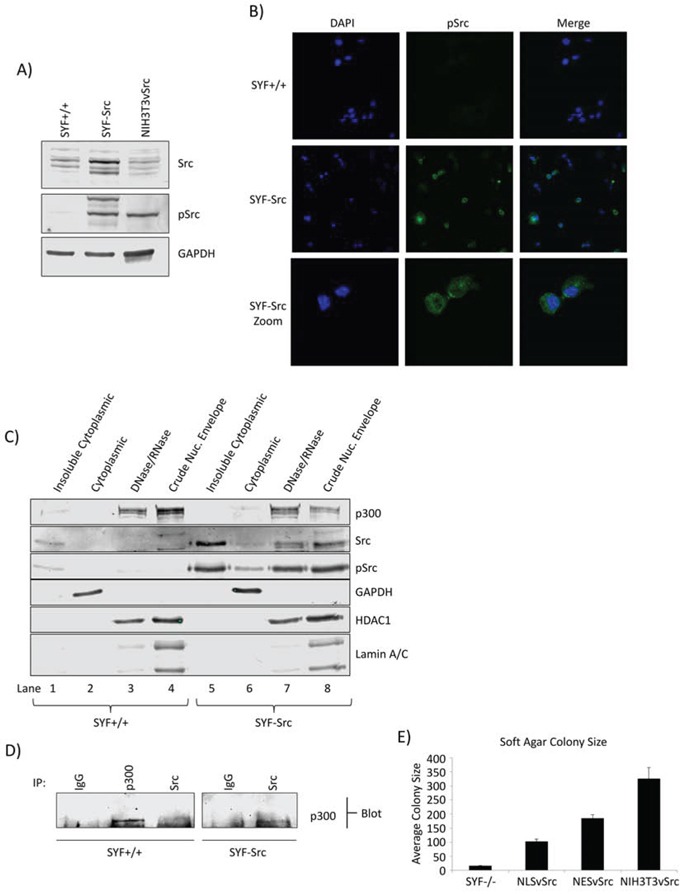
Src nuclear localization and association with p300 in MEF cells **A.** Phospho-Src and Src immunoblots in SYF MEF lines and NIH3T3vSrc cells. Immunoblots of total and pSrc levels in SYF+/+ and SYF-Src cells compared to NIH3T3vSrc. Total Src antibody recognizes c-Src only. **B.** Immunofluorescent staining of pSrc in SYF+/+ and SYF-Src cells. Cells on coverslips were fixed and stained with anti-pSrc antibody or DAPI (4′,6-diamidino-2-phenylindole). Images were acquired at 63X magnification using identical settings and are representative of >5 fields in three independent experiments. **C.** Subcellular fractionation and localization of p300, pSrc, and Src in SYF+/+ and SYF-Src cells. Immunoblots of p300, Src, pSrc, GAPDH (soluble cytoplasmic protein), HDAC1 (nuclear protein), and lamin A/C (nuclear lamina) in the designated subcellular fractions of SYF+/+ and SYF-Src MEFs. DNase/RNase treated nuclei were extracted with radioimmunoprecipitation assay (RIPA) buffer to produce the crude nuclear envelope fraction. **D.** Src associates with p300 in the SYF-Src cells. Immunoblotting analysis of Src or p300 immunecomplexes from SYF+/+ and SYF-Src cell nuclear extracts probing for p300. **E.** Soft agar colony sizes of SYF−/−, SYF−/−NLSvSrc, SYF−/−NESvSrc, and NIH3T3vSrc cells. The designated cells were grown in soft agar until large colonies were visible, which were stained, and >15 images were taken for each condition. Colony pixel diameter was quantified and plotted; results represent mean ±SEM of three independent experiments. Positions of proteins in gel are labeled. Data are representative of two to three independent experiments.

We next used the SYF group of mouse embryonic fibroblast (MEF) lines, wild-type MEFs (SYF+/+), Src-, Yes-, and Fyn-null MEFs (SYF−/−), and counterpart overexpressing exogenous c-Src in the SYF−/− background (SYF-Src) [24], as a second model to further explore/compare the nuclear Src signaling between cells that exhibit abnormal Src function (via exogenous Src over-expression) and cells of the same type that have normal levels. It is known that c-Src overexpression induces a degree of Src kinase activation associated with a modest growth in soft agar [25], which was confirmed using the SYF-Src cells, compared to the wild type SYF+/+ cells and using NIH3T3vSrc as positive control (Figure 2A, pSrc, Figure S2A). Given the differential Src activation in these cells, we speculated that there may be differential localization of the protein. Immunofluorescence with confocal microscopy imaging shows Src to be detectable throughout the cell in both SYF-Src and wild-type SYF+/+ cell lines, including the nucleus (Figure S2B). However, pSrc (active) was only detectable in the nuclei of the SYF-Src cells (Figure 2B). These results were confirmed by biochemical fractionation, with the distribution of active Src in the nuclear compartments of SYF-Src cells mirroring that observed in the Panc-1 cells (Figure 2C, last two lanes). *In vitro* nuclear kinase assay also showed that the treatment with dasatinib suppresses the number of phosphotyrosine proteins induced in the SYF-Src cell line, but not the wild-type SYF+/+ cells (Figure S2C, dasatinib, see arrows), suggesting that Src activity is enhanced in the SYF-Src cells, where it functions as a nuclear protein tyrosine kinase. Co-immunoprecipitation and immunoblotting analysis showed association between Src and p300 in the nuclear lysates of SYF-Src, but not the wild-type SYF+/+ cells (Figure 2D).

To further study the role of nuclear-targeted Src, we prepared vSrc constructs with a classical NLS or NES fused to the C-terminus, NLSvSrc and NESvSrc, respectively. The N-terminus of Src is known to contain membrane-targeting domains and a critical myristoylation sequence that strongly affect its signaling activity within cells [26]. Because of this, N-terminal fusion disrupts important functions of the protein, and C-terminal fusion is preferred for Src studies. The v-Src constructs (NLSvSrc and NESvSrc) were transfected into SYF−/− cells to generate a stable pool of SYF−/−NLSvSrc and SYF−/−NESvSrc cells expressing this construct, and the pSrc localization was verified by confocal microscopy (Figure S3A). It is known that vSrc with an NLS fused to the N-terminus does not induce morphological transformation [27], which was confirmed in our study using the SYF−/−NLSvSrc cells, compared to NIH3T3vSrc cells, while the SYF−/−NESvSrc cells appeared morphologically transformed (Figure S3B). Interestingly, both the SYF−/−NLSvSrc and NESvSrc cells were able to form colonies in soft agar. However, colonies from both of these cells were considerably smaller than those formed by NIH3T3vSrc cells (Figure 2E, Figure S3C). Altogether, our results indicate that active nuclear Src is present in PDAC and MEF cells over-expressing c-Src, and further that its association with p300 is evident in both PDAC and MEF background that over-expressed active Src. Results also demonstrate a clear function of nuclear Src in the MEF background.

### p300 is tyrosine phosphorylated in a Src-dependent manner

**Figure 3:**
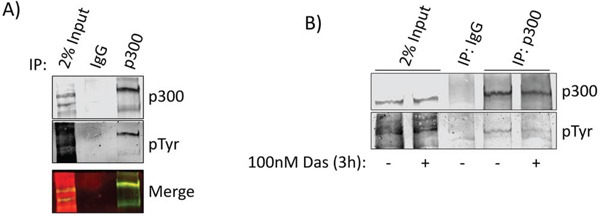
p300 is tyrosine phosphorylated in a Src-dependent manner **A.** Nuclear extracts from Colo-357 cells were subjected to immunoprecipitation with p300 antibody and probed simultaneously for p300 and phosphotyrosine (pTyr). Bands were imaged in different channels using the LiCor Odyssey CLx to verify overlap (merge). **B.** Colo-357 cells were treated with 100 nmol/L dasatinib or DMSO (control) as indicated. Nuclear lysate preparations were subjected to immunoprecipitation using anti-p300 antibody and immunoblotting analysis using general pTyr and p300 antibodies. Positions of proteins in gels are labeled. Control lanes (−) represent cells treated with 0.1% DMSO. Results represent three independent experiments.

While p300 is known to be serine phosphorylated at multiple residues [16, 17], there are no reports in the literature describing tyrosine phosphorylation of the protein. Src association with p300 led us to investigate whether p300 undergoes tyrosine phosphorylation. Immunoprecipitation of p300 and Western blotting analysis for general phosphotyrosine revealed tryosine phosphorylation of p300 in the PDAC cell line, Colo-357 (Figure 3A), which was suppressed when Colo-357 cells were treated with the Src inhibitor, dasatinib (Figure 3B), suggesting tyrosine phosphorylation of p300 is dependent on Src tyrosine kinase activity. We detected similar results in Panc-1 cells when immunoprecipitating either p300 or general phosphotyrosine and probing with the inverse antibody (Figure S4A, S4B). These results are the first to show a Src-dependent tyrosine phosphorylation of p300 in PDAC cells.

### Nuclear Src and p300 associate with HMGA2 and SMYD3 gene promoters and regulate their expression in pancreatic cancer cells and MEFs

**Figure 4:**
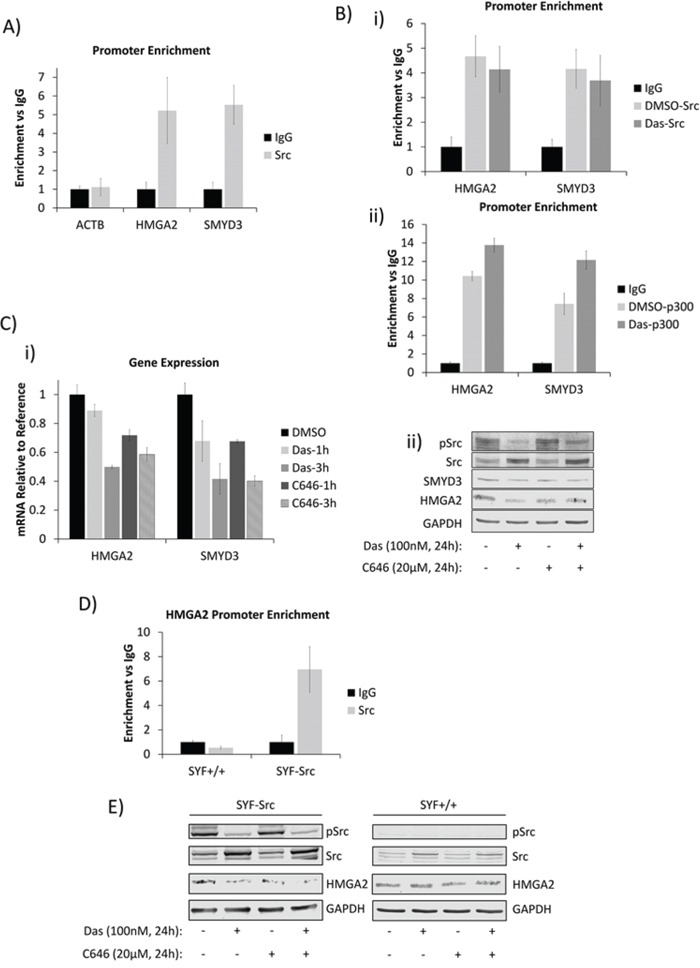
Nuclear Src and p300 associate with HMGA2 and SMYD3 gene promoters and regulate their expression in PDAC and MEF cells **A and B.** Chromatin-immunoprecipitation (ChIP) and quantitative polymerase-chain reaction (qPCR) analyses of Src-associated genes in Panc-1 cells and the effects of dasatinib. (A) Untreated Panc-1 cells were subjected to ChIP analysis using anti–Src antibody and qPCR to quantify the immune-complex-associated ACTB, HMGA2, or SMYD3 promoters. Pre-immune IgG was used as control. (B) Panc-1 cells were treated for three hours with 100 nmol/L dasatinib or untreated (DMSO) and subjected to ChIP using anti-Src (i), anti-p300 (ii), or control IgG antibodies as indicated. Antibody-associated eluates were analyzed by qPCR using primers spanning the promoter regions of the indicated genes. PCR signals were normalized to individual sample inputs and compared to IgG control and plotted as enrichment vs IgG. **C.** Analysis of HMGA2 and SMYD3 expression in Panc-1 cells. Panc-1 cells were treated with 100 nmol/L dasatinib, 20 μmol/L C646, or 0.01% DMSO (control) for the indicated times. Total RNA was extracted from cells for mRNA expression analysis (i), or whole-cell lysates were prepared for immunoblotting analysis of HMGA2 or SMYD3 expression (ii). **D.** Promoter enrichment as detected by ChIP-qPCR of Src at the mouse HMGA2 promoter in the SYF+/+ and SYF-Src cell lines. PCR reactions were performed using specific primers for the mouse HMGA2 promoter region analogous to the Src-associated region in the human HMGA2 promoter. Data are normalized to individual inputs and are expressed as enrichment vs IgG. All qPCR data represent mean ±SEM of three independent experiments. **E.** Immunoprobes of Src, pSrc, HMGA2 or GAPDH from whole-cell lysates prepared from SYF+/+ and SYF-Src cells treated with 100 nmol/L dasatinib or 20 μmol/L C646 for 24 hours. Control (−) lanes represent cells treated with 0.05% DMSO. Data are representative of three independent experiments.

We were interested in identifying genes regulated by nuclear Src. In order to define a putative list of targets, we performed chromatin immunoprecipitation coupled to human promoter microarray (ChIP-chip) to determine the promoters occupied by Src in Panc-1 cells. The raw gene list was filtered by only including genes with known function in PDAC and further by focusing only on genes associated with migration and invasion, processes which are known to be mediated by Src activity. This led to the identification of HMGA2 and SMYD3 genes, which were first confirmed by chromatin immunoprecipitation analysis for specific Src association with their promoters using primers corresponding to the ChIP-chip peaks within their promoter regions, compared to primers for the irrelevant beta-actin promoter as control (Figure 4A). Given that Src and p300 are associated in these cells and that p300 is tyrosine phosphorylated in a Src-dependent manner, we asked whether Src inhibition could disrupt Src or p300 association with these promoters. Inhibition of Src activity by dasatinib has no effect on the Src and p300 association with the gene promoters (Figure 4B). By contrast, Src inhibition by dasatinib or p300 inhibition by C646 [28] decreased both the mRNA and protein levels of HMGA2 and SMYD3 (Figure 4C). These results together suggest that the enzymatic activities of both Src and p300 contribute to the regulation of the target genes, but are not essential for their association with the target gene promoters. Critically, combined inhibition did not show an additive inhibitory effect on the protein expression of the target genes (Figure 4C(i)), suggesting a convergence of signaling in the regulation of these genes.

To attempt to rule out the canonical membrane-initiated signaling to p300, we probed the induction of the extracellular signal-regulated kinase (ERK) and phosphatidylinositide 3-kinases (PI3K)/AKT pathways, which are known to signal to p300 and are influenced by Src [16, 17] and observed no changes in response to Src or p300 inhibition at a time point when HMGA2 and SMYD3 expression are inhibited (Figure S5A). Moreover, ChIP studies also showed Src to be associated with the HMGA2 promoter in the SYF-Src cells, but not in the SYF+/+ line (Figure 4D). Inhibition of Src or p300 decreased the expression of HMGA2 in SYF-Src cells, while having no effect on HMGA2 expression in the SYF+/+ or SYF−/− cells (Figure 4E, Figure S5B). Together, these data indicate that nuclear active Src in conjunction with p300 is specifically associated with HMGA2 and/or SMYD3 gene promoters and regulates their expression in a Src-kinase-dependent manner in PDAC and MEF cells. While these results do not rule out involvement of other SFKs, they do demonstrate that Src is able to induce these changes alone.

### Src and p300 signaling axis supports tumor cell migration and invasiveness in a nuclear Src-dependent manner

We next investigated the effects of Src and p300 inhibition on the PDAC cell phenotype. Treatment with the Src inhibitor, dasatinib, or the p300 inhibitor, C646, had no effect on the proliferation of the three PDAC lines, Panc-1, Colo-357 and BxPC-3 (Figure S5C, D). The effect of inhibition of either protein on cell proliferation was moderate in the SYF-Src cell line, minimal in the wild type SYF+/+ cells, and none in SYF−/− cells (Figure S5E). These results are consistent with the clinical trials of Src inhibitors in advanced PDAC cases, which showed no therapeutic benefit [29], and together suggest that Src does not significantly contribute to PDAC cell survival.

**Figure 5:**
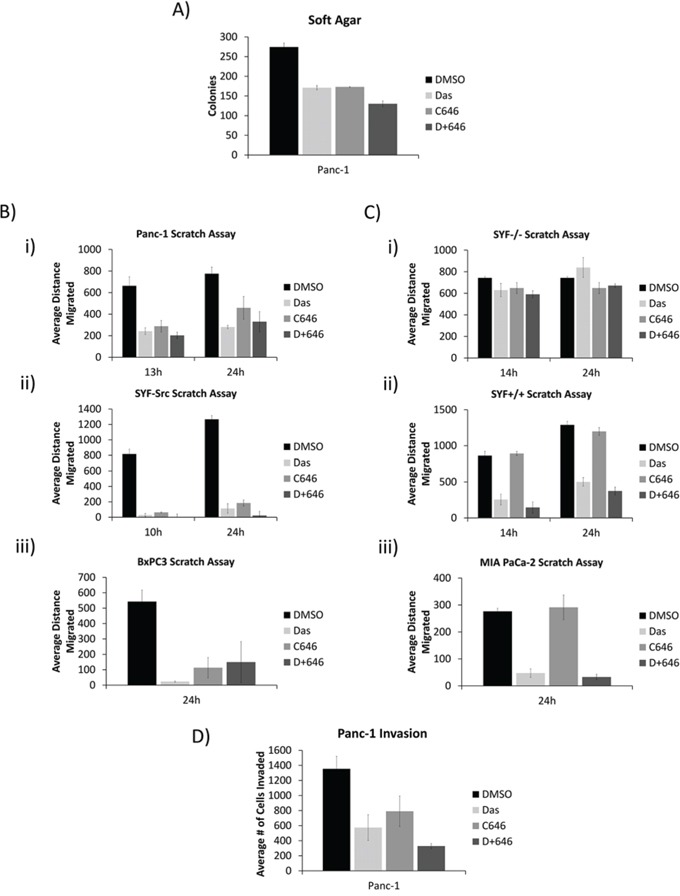
Src and p300 signaling axis affects tumorigenic properties in a nuclear Src-dependent manner **A.** Effect of dasatinib and C646 on soft agar growth of Panc-1 cells. Panc-1 cells growing in soft agar were overlaid with medium containing 100 nmol/L dasatinib and 20 μmol/L C646. Colonies were quantified using AlphaView and expressed as mean ±SEM of three independent experiments. **B and C.** Confluent monolayers of Panc-1 (B-i), SYF-Src (B-ii), BxPC3 (B-iii), SYF−/− (C-i), SYF+/+ (C-ii), and MIA PaCa-2 (C-iii) cells in culture were untreated (DMSO) or treated with 100 nmol/L dasatinib or 20 μmole/L C646 as indicated. Monolayers were scratched and cells were allowed to migrate into the denuded area for the indicated times. Distance migrated was measured as the change in scratch width at the indicated time point. Data are represented as mean ± SEM of three individual experiments. **D.** Panc-1 cell invasion studies and the effects of dasatinib and C646. Panc-1 cells were seeded onto matrigel-coated invasion wells and allowed to invade for 24 hours into the lower chamber in the presence of 100 nmol/L dasatinib or 20 μmol/L C646. Invading cells were counted and represented as mean ± SEM of three independent experiments. All samples contained a final DMSO concentration of 0.05%.

On the other hand, in soft agar studies, inhibition of Src, p300, or both suppressed anchorage-independent growth of Panc-1 cells (Figure 5A). Scratch (in vitro migration) assay further showed the inhibition of Src or p300 activity suppressed the migration of SYF-Src, Panc-1, and BxPC-3 cells (Figure 5B, Figure S6). By contrast, MIA PaCa-2, SYF+/+, and SYF−/− cells, which did not exhibit Src/p300 dependence were differentially sensitive to Src inhibition in the migration assay, but all were completely refractory to p300 inhibition (Figure 5C, Figure S7). Of note, cells that showed p300-dependency in this assay (SYF-Src, Panc-1, and BxPC-3) were quicker to fill the scratched area than those lines that appeared to be p300-independent. These results suggest that the nuclear Src/p300 axis functions separately from the canonical membrane-associated Src signaling to support the migration of cells that harbor active Src. Inhibition of Src or p300 activity also decreased the invasive properties of Panc-1 cells (Figure 5D).

### Nuclear Src activation and association with HMGA2 and SMYD3 gene promoters are observed in patient samples of PDAC

**Figure 6:**
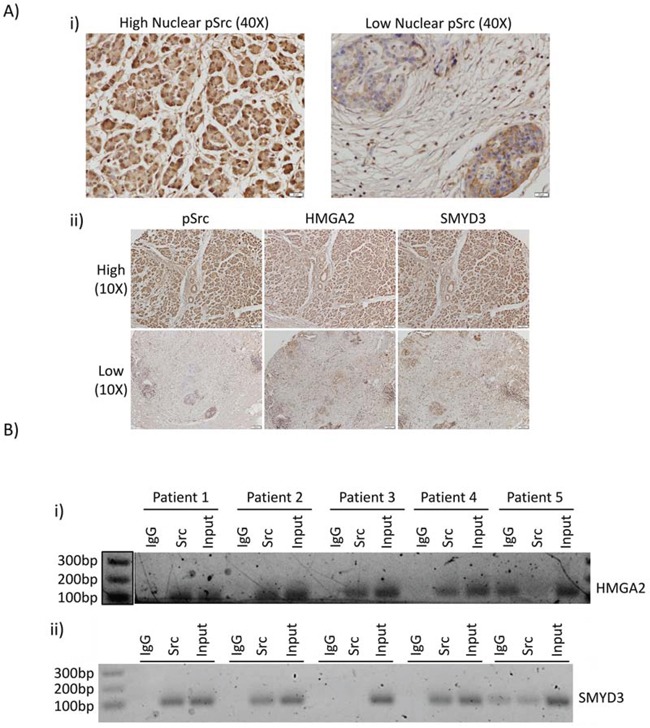
Nuclear Src activation and association with HMGA2 and SMYD3 gene promoters are observed in patient samples of PDAC **A** Immunohistochemistry of human PDAC tumor samples. Human PDAC slides were immunostained for pY416Src (i) and (ii, left panels), HMGA2 (ii, middle panels), or SMYD3 (ii, right panels). Images were acquired at high (40X) magnification for pSrc (i), or low magnification (10X) of the same tumors for pSrc, HMGA2, or SMYD3 (ii). **B.** Pathology tissue-Chip (PAT-ChIP) of human PDAC samples derived from banked formalin-fixed paraffin-embedded tissues. Samples were subjected to chromatin immunoprecipitation assay using anti-Src antibody or IgG as control and amplified by PCR using specific primers for the HMGA2 (i) and SMYD3 (ii) promoters. Correct PCR product size is verified by DNA ladder.

We extended the studies to investigate nuclear expression of active Src, HMGA2, and SMYD3 expression in human PDAC tumor samples. Active nuclear Src has been previously identified in breast cancer [30] and colon cancer [31], though this localization has generally not been examined closely in other tumor types. Immunohistochemistry analysis showed detectable nuclear and non-nuclear active Src in a human PDAC tissue microarray (Figure 6A, 40X magnification). Higher HMGA2 and SMYD3 expression were detected in the tumors harboring high nuclear pSrc compared to the tumors lacking active nuclear Src (Figure 6A(ii), representative data). In order to determine whether Src association with the HMGA2 and SMYD3 promoters is maintained in the patients’ tumors *in vivo*, we performed ChIP on samples derived from formalin-fixed paraffin embedded (FFPE) tumor blocks utilizing the newly developed PAT-ChIP method [32]. Src was found to be associated with the HMGA2 promoter in four out of five FFPE blocks and with the SMYD3 promoter in three out of five blocks (Figure 6B). These results show that active nuclear Src is detectable within patient tumor samples of PDAC and is associated with the HMGA2 and SMYD3 promoters.

## DISCUSSION

**Figure 7:**
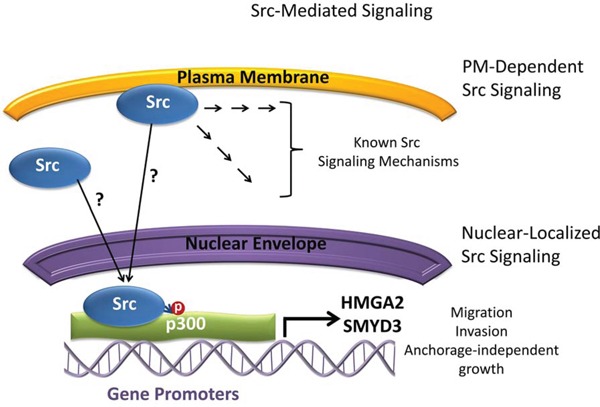
Model depicting Src signaling and the relationship with p300, HMGA2, and SMYD3 in Panc-1 cells Plasma membrane (PM)-dependent induction of Src signaling and the transduction of signal cascades into the cell are well studied. Nuclear translocation and localization of Src from the plasma membrane or cytosol occur by unknown mechanisms and result in Src interacting with p300 and mediating p300 tyrosine phosphorylation. Nuclear Src and p300 complex associates with the promoter regions of the HMGA2 and SMYD3 genes and regulate their expression. The functions of nuclear Src/p300 signaling axis regulate the migratory, invasive, and anchorage-independent growth properties of cells. ?, undetermined; →, signal transduction steps, P, phosphorylation.

In addition to Src overexpression and hyperactivation in PDAC [5], our studies and others show active Src in the nuclear compartment of cancer cell lines and patient tumors [10–13], comparable to the other tyrosine kinases reported to be functionally active in the nucleus [14, 33–35]. Notably, ChIP-chip and IP-MS studies found nuclear Src and p300 to form a novel complex in Panc-1 cells, which is associated with the promoters of genes, including the pro-migratory genes HMGA2 and SMYD3. Nuclear Src was similarly detected to be associated with HMGA2 and SMYD3 promoters in clinical PDAC tissue samples. Studies using small molecule inhibitors suggest the enzymatic activities of both Src and p300 converge for the regulation of HMGA2 and SMYD3 expression (Figure 7).

We note that neither Src nor p300 exhibits any sequence-specific DNA binding activity, and that the binding of the two proteins to target genes are likely both chromatin context and cell-type dependent. Accordingly, immunoblotting analysis detected basal expression of HMGA2 in all three MEF cell lines investigated irrespective of Src status (SYF+/+ (wild type), SYF−/− (knockout), and SYF-Src (over-expressing exogenous Src in the knockout background)). While the basal level of HMGA2 protein was similar, the association of Src with the HMGA2 promoter is only observed in the SYF-Src line exhibiting a degree of Src activation. Further, Src/p300 complexation and the regulation of HMGA2 expression by Src/p300 complex are only maintained in the SYF-Src cells, suggesting a switch in the control of HMGA2 regulation to Src/p300-dependency when Src is active. Contrastingly, neither SMYD3 expression nor Src association with the SMYD3 promoter was detected in any of the MEF cell lines (data not shown). These results together suggest that nuclear Src/p300 signaling is associated with accessible or active chromatin to perform regulatory functions and that HMGA2 is likely a major mediator of downstream events from the Src/p300 signaling axis to support the altered phenotype.

The role of p300 in PDAC is largely unexplored, although its function in other tumor types has begun to be described in more detail. In particular, p300 is reported to be a key mediator of oncogenic hormone receptor signaling in hormone receptor positive breast and prostate cancers [36] whereby it promotes the expression of estrogen or androgen receptors, respectively [37, 38]. Studies with specific p300 inhibitors have also implicated the protein as a mediator of the oncogenic properties of melanoma [28], acute myeloid leukemia (AML) [39], neuroblastoma [40], breast cancer [41], and prostate cancer [42]. The current studies show for the first time that p300 is involved in the migratory and metastatic behavior of pancreatic cancer cells. In this context, p300 is tyrosine phosphorylated in a Src-dependent manner. In consideration of the reported significant roles of both Src and p300 in hormone receptor signaling [36, 43], current studies raise the possibility that the novel Src/p300-dependent signaling occurs in hormone receptor positive tumors, such as breast and prostate cancers. This is consistent with the report that high nuclear pY416 Src is associated with significantly better response to tamoxifen ER antagonist [30] and that active nuclear Src contributes to the dependency on ER signaling of the breast cancer phenotype.

While clinical trials of Src inhibitors failed to show efficacy in patients with advanced metastatic disease [29, 44], present data may offer some explanation for this failure in indicating that Src and p300 participate in the migratory and invasive properties of PDAC cells rather than survival signaling. In addition, while the ineffectiveness of Src inhibitors in advanced PDAC may seem to cast doubt on the relevance of Src as a target in PDAC, others have contended that clinical and preclinical data supports Src inhibition under specifically-defined circumstances, but not as a general therapeutic agent for advanced disease [45]. Our data suggest that therapies targeting Src/p300 signaling could be efficacious early intervention options for aggressive primary tumors in which both Src and p300 are implicated in order to prevent primary tumor metastasis.

Altogether, current studies provide new insights into the functional roles of nuclear Src in PDAC (Figure 7). The identification of HMGA2 and SMYD3 as targets of Src/p300 signaling provides additional novel mechanisms by which Src likely mediates cellular migratory and invasive properties, while also showing for the first time that p300 participates in the regulation of the migratory and invasive potential of PDAC cells. Critically, the observation that the Src/p300 signaling axis depends on the enzymatic activities of the two proteins signifies a novel signaling target for therapeutic intervention in PDAC.

## MATERIALS AND METHODS

### Chemicals and antibodies

The Src inhibitor dasatinib (ChemieTEK, Indianapolis, IN) and the p300 inhibitor C646 (Sigma Aldrich, St. Louis, MO) were purchased from the designated sources. Antibodies used were as follows: Src (B-12), p300 (C20), GAPDH (6C5), β-Tubulin (C10), Grim19, (Santa Cruz Biotechnology, Santa Cruz, CA), Src (36D10), pY-416 Src (2101), HMGA2 (D1A7), SMYD3 (D2Q4V), Lamin A/C (#2032), Calnexin (C5C9), HDAC1 (10E2), Histone H3 (D1H2), p-TYR-100 (Cell Signaling Technology, Danvers, MA), pY418 Src (92633), p300 (507) (Novus Biologicals, Littleton, CO), HMGA2 (AF3184) (R&D Systems, Minneapolis, MN), and SMYD3 (ab16027) (Abcam, Cambridge, UK). Secondary antibodies used were Donkey-anti-Rabbit 800CW and Goat-anti-Mouse 680LT (Licor, Lincoln NE).

### Cell culture

The PDAC cell lines Panc-1, BxPC3, Colo-357, and MIA PaCa-2 have all been reported [14]. The Src^−/−^, Yes^−/−^, Fyn^−/−^ cell line (SYF−/−), the triple-null cells overexpressing c-Src (SYF-Src), and the wild type mouse embryonic fibroblast (MEF) cells (SYF+/+) were generous gifts of Dr. Leda Raptis, Queens University, Kingston, Ontario, and have been previously reported [24]. Mouse fibroblasts transformed by viral Src (NIH3T3vSrc) have also been reported [46]. All cells were grown in Dulbecco's modified Eagle's medium supplemented with 10% heat-inactivated fetal bovine serum (FBS) and 1X PenStrep (Gibco, Life Technologies, Carlsbad, CA), with some exceptions. MIA PaCa-2 cells were grown in identical medium supplemented with an additional 2.5% horse serum. NIH3T3vSrc cells were cultured in DMEM containing 5% bovine calf serum (BCS) and 1X PenStrep.

### Plasmids and vector construction

Sequences corresponding to the nuclear export signal (NES) of protein kinase A inhibitor alpha (PKIα) (LALKLAGLDI-Stop) or the nuclear localization signal (NLS) of the SV40 Large T antigen (PKKKRKVE-Stop) were inserted between the NotI and XbaI cut sites of the pTracer-CMV vector to yield pTracer-NES and pTracer-NLS, respectively. Next, vSrc cDNA from the pM-vSrc vector [46] was subcloned, excluding the stop codon, into the above vectors using the EcoRI and NotI cut sites. The final products yielded C-terminally tagged NES-vSrc or NLS-vSrc.

*Western Blotting* was performed as previously described [47, 48]. Specific primary antibodies used for immunoblotting were Src (B-12), p300 (C20) (Santa Cruz), pY-416 Src (2101), HMGA2 (D1A7), and SMYD3 (D2Q4V) (Cell Signaling). Nitrocellulose membranes were scanned using the Licor Odyssey CLx Infrared Imaging Scanner (Lincoln, NE).

### Immunofluorescence

Immunofluorescent staining of cells was performed as described previously [48]. Additional information is available in Supplementary Materials and Methods.

### Cellular and subnuclear fractionation

Plated cells were briefly swelled in hypotonic buffer (10 mmol/L HEPES, pH 7.9, 1.5 mmol/L MgCl_2_, 10 mmol/L KCl), then membranes were solubilized in hypotonic buffer + 0.2% Nonidet P (NP)-40. Cytosolic and nuclear fractions were separated by brief centrifugation at 800g for five minutes. The supernatant was collected and further centrifuged at 17,000g for 15 minutes to obtain the soluble cytoplasmic supernatant and the insoluble cytoplasmic pellet. The nuclear pellet was washed once in hypotonic buffer + 0.2% NP-40 and centrifuged at 800g for five minutes. The nuclear pellet was either resuspended in immunoprecipitation (IP) buffer (25 mmol/L Tris, pH 7.4, 150 mmol/L NaCl, 1 mmol/L EDTA, 5% glycerol, 1% NP-40) and centrifuged at 17,000g for 15 minutes for use in immunoprecipitation studies, or further processed for subnuclear fractionation as previously described [22], with minor alterations. Details are provided in Supplementary Materials and Methods.

### Nuclear kinase assay

Nuclei were first isolated in the absence of Na_3_VO_4_. Equal volumes of isolated nuclei were resuspended in ice cold kinase activity buffer (50 mmol/L Tris-HCl, pH 7.5, 10 mmol/L MgCl_2_, 2.5 mmol/L MnCl_2_, 10 mmol/L DTT, 1 mmol/L ATP) in the presence or absence of 100 nmol/L dasatinib and 1 mmol/L Na_3_VO_4_. Samples were then incubated at 37°C for 30 minutes and then placed on ice. Nuclear protein was harvested with radioimmunoprecipitation assay (RIPA) buffer (25 mmol/L Tris-HCl, pH 7.5, 150 mmol/L NaCl, 1% NP-40, 1% sodium deoxycholate, 0.1% SDS), and samples of equal total protein were subjected to SDS-PAGE and immunoblotting analysis probing for general phosphotyrosine.

### Immunoprecipitation

Immunoprecipitation was performed as described previously, with some modification [47, 48]. Briefly, protein was extracted from nuclear pellets with IP buffer and diluted to 50 mmol/L NaCl, then pre-cleared with IgG and protein A/G PLUS agarose beads (Santa Cruz). Cleared lysates were incubated with specific antibody or IgG overnight with agitation at 4°C. Immune complexes were precipitated with protein A/G PLUS beads, washed, and subjected to SDS-PAGE and immunoblotting analysis. Specific antibodies used for antigen capture in IP studies were Src (B-12), p300 (C20) (Santa Cruz), and p-TYR-100 (Cell Signaling).

### Immunoprecipitation-mass spectrometry

For IP-MS, 3 × 10^8^ cells were subjected to immunoprecipitation using Src antibody or IgG control. Immune-bead complexes were washed, boiled, and further subjected to precipitation using trichloroacetic acid (TCA). Dried pellets were sent to Taplin Biological Mass Spectrometry Facility (Boston, MA) for peptide analysis.

### Chromatin immunoprecipitation (ChIP)

These studies were performed as previously described [48]. Nuclei from 1.2 × 10^7^ cells were sonicated to yield an average chromatin fragment length of 200-700bp. Chromatin samples were also subjected to ChIP-chip analysis, as described in detail in Supplementary Materials and Methods.

*Quantitative PCR* was performed as described previously [48]. Primers used and additional information are available in Supplementary Materials and Methods.

### Soft agar colony formation assay

Cell growth in soft agar was performed as previously described [46, 48]. After solidification of upper agar layer containing cells, medium containing inhibitors or DMSO (control) was overlaid. Cells were allowed to grow for three weeks until colonies were visible. For size quantification, ≥15 images were taken for each cell line in three independent experiments, then colony pixel diameter was measured and reported as mean ±SEM.

### Wound healing assay

Scratch migration assays were performed as previously reported [48]. Further details are available in Supplementary Materials and Methods.

### Invasion chamber assay

Cell invasion assay was performed and quantified as previously described [48, 49].

### Immunohistochemistry (IHC)

Commercial tissue microarray (TMA) (Pan03-001; US Biolab Corp., Gaithersburg, MD) was examined by IHC staining using standard protocols as previously described [50]. Antibody and procedure details are available in Supplementary Materials and Methods.

### Pathology tissue (PAT)-ChIP

ChIP from patient formalin-fixed paraffin-embedded (FFPE) tissues was performed precisely as reported [32] with no modification. PCR bands were resolved on a 1% agarose gel and visualized with ethidium bromide.

## SUPPLEMENTARY DATA FIGURES AND TABLE



## Abbreviations

PDAC: pancreatic ductal adenocarcinoma
HAT: histone acetyltransferase
MEF: mouse embryonic fibroblasts
SYF: Src-, Yes-, and Fyn-null MEF background (SYF−/−) and the Src-overexpressing (SYF-Src) or wild type (SYF+/+) counterparts
NLSvSrc and NESvSrc: SYF−/− cells stably expressing vSrc with a C-terminal NLS or NES respectively
DMSO: dimethyl sulfoxide
AR: androgen receptor
ER: estrogen receptor
FBS: fetal bovine serum
qPCR: quantitative polymerase chain reaction
ChIP: chromatin immunoprecipitation
MS: mass spectrometry
IHC: immunohistochemistry
