# Supplementary material for: A novel nuclear Src and p300 signaling axis controls migratory and invasive behavior in pancreatic cancer
Source: Oncotarget. 2015 Dec 17;7(6):7253–67. doi: 10.18632/oncotarget.6635 (PMC4872783; doi:10.18632/oncotarget.6635)
Supplement: Supplementary file 1 [file oncotarget-07-7253-s001.pdf]

SUPPLEMENTARY DATA

MATERIALS AND METHODS

Reagents and antibodies

Additional antibodies used in these studies were as follows: pS2448mTOR (D9C2), mTOR (7C10), pS473 AKT (D9E), AKT (9272), pERK1/2 (D13.14.4E), ERK1/2 (9102) (Cell Signaling Technologies, Danvers, MA).

Immunofluorescence (IF) and confocal microscopy

IF was performed as previously described with some modification [1, 2]. Briefly, cells were seeded onto coverslips, and then fixed with 4% paraformaldehyde in phosphate buffered saline (PBS) for ten minutes at room temperature. After fixation, cells were permeabilized by incubation with PBS+0.25% Triton X-100 for ten minutes. Cells were washed with PBS+0.1% Tween-20 (PBS-T) and then blocked with PBS-T + 0.2% BSA. One set of coverslips was incubated with primary antibodies for one hour at room temperature, then washed three times in PBST for five minutes each. Another set of coverslips was processed in parallel without primary antibody as a control to determine non-specific background staining. Both sets of coverslips were incubated with secondary antibody for one hour, washed with PBST three times for five minutes each, and mounted onto microscope slides with Fluoromount-G + DAPI (SouthernBiotech, Birmingham, AL) and imaged on the Leica TCS-SP5 microscope system. Primary antibodies used in these studies were anti-Src (36D10) (Cell Signaling), p418Src (NB100–92633), and p300 (NB100–507) (Novus Biologicals, Littleton, CO). Secondary antibodies were Alexa 488 goat-anti-rabbit and Alexa 594 goat-anti-mouse (Molecular Probes, Eugene, OR).

Subnuclear fractionation

Fractionation was performed as previously reported [3] with some exceptions. Pelleted nuclei were suspended in 0.5mL lysis buffer (0.1 mmol/L $MgCl_2$, 1 mmol/L DTT, 10 µg/mL RNase A, 10 µg/mL DNase I, and cocktails of protease and phosphatase inhibitors (Sigma Aldrich, St. Louis, MO)) and transferred into a 15mL centrifuge tube. Next, 2mL extraction buffer (10% sucrose, 20 mmol/L triethanolamine, pH 8.5, 0.1 mmol/L $MgCl_2$, 1 mmol/L DTT, and protease and phosphatase inhibitors) was added dropwise, while gently vortexing. Nuclei were incubated at room temperature for 30 minutes to digest RNA and accessible chromatin. Next, 7mL of ice-cold sucrose cushion buffer (30% sucrose, 20 mmol/L triethanolamine, pH 7.5, 0.1 mmol/L $MgCl_2$, 1 mmol/L DTT, and protease and phosphatase inhibitors) was underlaid and the tube was centrifuged in a tabletop centrifuge at 750g for 10 minutes. The upper layer supernatant was removed and labeled as “DNase/RNase fraction,” representative of the soluble nucleoplasmic material liberated by digestion of accessible chromatin and RNA. Next, the underlayment was removed and the pellet was resuspended in 0.5mL extraction buffer (10% sucrose, 20 mmol/L triethanolamine, pH 7.5, 0.1 mmol/L $MgCl_2$, 1 mmol/L DTT, and protease and phosphatase inhibitors), to which was added 0.25mL extraction buffer containing 0.3mg/mL heparin dropwise, while gently vortexing. Seven milliliters of sucrose cushion buffer was again underlayed and the samples were centrifuged at 750g for 15 minutes. The supernatant was removed and labeled as “Heparin extract,” representative of the DNAse-inaccessible nucleoplasmic heterochromatin. The pellet was lysed in radio immunoprecipitation assay (RIPA) buffer (25 mmol/L Tris-HCl, pH 7.5, 150 mmol/L NaCl, 1% NP-40, 1% sodium deoxycholate, 0.1% SDS) and labeled “Nuclear envelope.” For “Crude nuclear envelope” preparation, the pellet following DNAse/RNase treatment was lysed in RIPA buffer and centrifuged at 17,000g for 15 minutes at 4°C.

Stable transfectant selection

Src, Yes, and Fyn-null MEF (SYF-/-) cells were first subjected to a Zeocin (Gibco) kill curve and a concentration of 200 µg/mL was determined to be optimal for selection. Cells were transfected with either pTracer-NLSvSrc or pTracer-NESvSrc using Lipofectamine 2000 (Invitrogen). After two days, medium containing 200 µg/mL Zeocin was added freshly every three to five days for approximately two weeks until colonies were visible. Cells from these colonies were harvested by trypsinization and seeded into new culture dishes as pools of either NLSvSrc or NESvSrc stable transfectants. These cells were then cultured in media containing 50 µg/mL Zeocin to maintain selective pressure.

Chromatin immunoprecipitation (ChIP)

ChIP assay was performed according to the Affymetrix Chromatin Immunoprecipitation Assay protocol and as previously described [4]. Briefly, 1.2 x 10^7 cells were fixed in 1% formaldehyde at room

temperature for seven minutes, and then fixation was quenched with 0.125M glycine for 10 minutes at room temperature. Cells were then washed three times with ice cold PBS and resuspended in lysis buffer for nuclear isolation. Nuclei were then sonicated (Omni International) at 50% power set to 50% pulse for shearing DNA to an average fragment size of 200–700bp. Lysates were then precleared with protein A/G agarose beads (Santa Cruz Biotechnology, Dallas, TX) for one hour at 4°C while rocking. Cleared lysates were incubated with anti-p300 (C-20), anti-Src (B12) antibody, or pre-immune mouse or rabbit antibody as control. Samples were incubated overnight at 4°C while rocking. Immune complexes were precipitated with protein A/G agarose beads and washed in buffers of increasing stringency. Beads were washed twice in Wash Buffer 1 (20 mmol/L Tris pH 8.0, 2 mmol/L EDTA, 1% Triton X-100, 150 mmol/L NaCl, and protease and phosphatase inhibitors), once each with Wash Buffer 2 (20 mmol/L Tris pH 8.0, 2 mmol/L EDTA, 1% Triton X-100, 0.1% SDS, 500 mmol/L NaCl, and protease and phosphatase inhibitors) and Wash Buffer 3 (10 mmol/L Tris pH 8.0, 1 mmol/L EDTA, 25 mmol/L LiCl, 0.5% NP-40, 0.5% Deoxycholate), and then twice in TE buffer (10 mmol/L Tris pH 8, 1 mmol/L EDTA). Samples were eluted and then treated with proteinase K (Pierce, Waltham, MA) at 55°C overnight to both digest protein and reverse crosslinks. DNA was purified with ChIP Spin Columns (Zymo Research Corp, Irvine, CA) and subjected to quantitative polymerase chain reaction (qPCR) analysis.

ChIP-on-chip assay and analysis

Purified DNA from ChIP samples was subjected to two rounds of manual linear amplification with adapter-linked random nonamer primers (GTTTCCCAGTCACGGTC$(N)_9$) using Sequenase (Affymetrix, Santa Clara, CA) reagents. After linear amplification, samples were exponentially amplified using Taq polymerase for 30 cycles with a 20%/80% ratio of dUTP/dTTP in order to incorporate uracil into the fragments. Amplified DNA was sent to the University of Hawaii Cancer Center Genomics Core Facility (Honolulu, HI) for uracil DNA Glycosylase fragmentation and labeling, and hybridization to the Human Promoter 1.0r Microarrays (Affymetrix). Once the arrays were hybridized and scanned, 'CEL' files containing raw intensity values were provided for analysis. 'CEL' files corresponding to each array were converted to 'TAG' files in the Affymetrix Tiling Analysis software suite. 'TAG' files were generated from raw data using quartile normalization with a target intensity of 250. Data were expressed with a Log2 signal scale and a -10Log10 *p*-value scale. Analysis of 'TAG' files was performed as a one-sided upper perfect match only using a bandwidth of 250. The *p*-value threshold was set to 23 (p=.005) with a maximum gap of 100bp and a minimum run of 200bp in order to exclude nonspecific hits from individual probes with artificially high signal. The analysis generated 'CHP' files, which could be viewed on the Affymetrix Integrated Genome Browser represented as *p*-value of enrichment of the specific IP vs the control IgG for each probe.

RTPCR and quantitative PCR

qPCR was performed as previously described [4] with some modification. For mRNA analysis, RNA samples were harvested using the PureLink RNA Mini kit (Ambion, Foster City, CA) according to the manufacturer's instructions, and reverse transcription was performed using the qScript cDNA Synthesis kit (Quanta Biosciences, Gaithersburg, MD) according to the manufacturer's instructions. Synthesized cDNA was analyzed by qPCR using a StepOnePlus thermocycler (Applied Biosystems Incorporated, Carlsbad, CA) with SYBR Select qPCR master mix (Applied Biosystems). HMGA2 and SMYD3 expression were normalized to the geometric mean of actin-β (ACTB) and $β_2$ microglobulin (β2M) expression within each treatment condition by the ΔΔ-Ct method. Samples were run in triplicate. Primers used in these studies were as follows: ACTB forward 5′-tgccgacaggatgcagaag-3′, reverse 5′-gccgatccacacggagtact-3′; β2M forward 5′-tacatgtctcgatcccacttaactat-3′, reverse 5′-agcgtactccaaagattcaggtt-3′; HMGA2 forward 5′-cagccgtccacatcagcccag-3′, reverse cttgcgaggatgtctcttcag-3′; SMYD3 forward 5′-tgttctccgtgtattggctttt-3′, reverse 5′-tttgcagcttttaaggcatttg-3.′

For DNA from ChIP samples, specific immune complexes and IgG samples were normalized by ΔΔ-Ct to the individual sample inputs to determine relative enrichment for each antibody. Relative enrichment for specific IP was then normalized to IgG and expressed as enrichment/IgG. Samples were run in triplicate. Primers used in these studies were as follows: Human ACTB promoter forward p5′-ggacatctcttgggcactga-3′, reverse 5′-gttgctctgaagtcggcaag-'3; human HMGA2 promoter forward 5′-tgtcattgataaagtgcaatatgagaa-3′, reverse 5′-tgccatacaggatgagaaaagc-3′; human SMYD3 promoter forward 5′-gcagatcacatgaaaaatgaacc-3′, reverse 5′-ttttcccagtggacactgttct-3′; mouse HMGA2 promoter forward 5′-ctggctgtcctggaactcac-3′, reverse 5′-aagatgacctttgccgcact-3.′ For all qPCR primer sets, PCR efficiency was > 90% and melt curve analysis and agarose gel electrophoresis confirmed a single product of the expected size.

Cell viability assay

CyQuant cell proliferation assay was performed as previously described [4, 5]. Cells were seeded in triplicate in 96-well plates and grown for either 72 or 48 hours in the presence of inhibitors. Initial cell density was determined such that the DMSO control would grow to < 90% confluence by the end time point.

Wound healing assay

Cells were seeded as confluent monolayers into 12-well plates in triplicate. Monolayers were scratched with a p10 pipet tip, then fresh medium containing 100 nmol/L dasatinib or 20 µmol/L C646 was added. Plates were then marked with a thin-tipped permanent marker to denote the imaging field across the scratch. Cells were imaged in phase contrast with the Zeiss Axiovert 200 microscope at 10X magnification immediately after the addition of inhibitor-containing media and noted as the zero hour time point. Cells were allowed to migrate for the indicated amount of time and imaged again at the same field marked on the plate.

Immunohistochemichal staining of tissue microarrays

Staining was performed as described previously [6]. Tissue microarray (TMA) was constructed from clinical samples obtained from a cohort of 62 cases. TMA was deparaffinized in xylene, rehydrated using graded percentages of ethanol followed by antigen retrieval with citric acid buffer (pH 6.0, 95°C for 20 minutes). The slides were treated with 3% hydrogen peroxide in water to block endogenous peroxidase activity. After 40 minutes blocking in 1% BSA in PBS (pH 7.4), slides were incubated overnight at 4°C with the following primary antibodies: anti-pSrc (Novus Biologicals, Littleton, CO, NB100–92633; rabbit polyclonal, dilution 1:100), anti-HMGA2 (R&D Systems, Minneapolis, MN, AF3184; goat polyclonal, dilution 1:100), or anti-SMYD3 (Abcam, Cambridge, UK, ab16027; rabbit polyclonal, dilution 1:200). Biotin-labeled goat anti-rabbit IgG (2 µg/ml in blocking buffer, Vector Laboratories, Burlingame, CA) or biotin-labeled rabbit anti-goat IgG (2 µg/ml in blocking buffer) was used as secondary antibody. Immunoreactive signals were amplified by formation of avidin-biotin peroxidase complexes and visualized using 3, 3′-diaminobenzidine (DAB). Nuclear counterstaining was conducted with hematoxylin.

REFERENCES

1. Zhao W, Jaganathan S and Turkson J. A cell-permeable Stat3 SH2 domain mimetic inhibits Stat3 activation and induces antitumor cell effects *in vitro*. J Biol Chem. 2010; 285:35855–35865.
2. Yue P, Zhang X, Paladino D, Sengupta B, Ahmad S, Holloway RW, Ingersoll SB and Turkson J. Hyperactive EGF receptor, Jaks and Stat3 signaling promote enhanced colony-forming ability, motility and migration of cis-platin-resistant ovarian cancer cells. Oncogene. 2011; 31:2309–2322.
3. Matunis MJ. Isolation and fractionation of rat liver nuclear envelopes and nuclear pore complexes. Methods. 2006; 39:277–283.
4. Yue P, Lopez-Tapia F, Paladino D, Li Y, Chen CH, Hilliard T, Chen Y, Tius MA and Turkson J. Hydroxamic acid and benzoic acid-based Stat3 inhibitors suppress human glioma and breast cancer phenotypes *in vitro* and *in vivo*. Cancer Res. 2015.
5. Zhang X, Yue P, Page BD, Li T, Zhao W, Namanja AT, Paladino D, Zhao J, Chen Y, Gunning PT and Turkson J. Orally bioavailable small-molecule inhibitor of transcription factor Stat3 regresses human breast and lung cancer xenografts. Proc Natl Acad Sci U S A. 2012; 109:9623–9628.
6. Zhang G, Gomes-Giacoia E, Dai Y, Lawton A, Miyake M, Furuya H, Goodison S and Rosser CJ. Validation and clinicopathologic associations of a urine-based bladder cancer biomarker signature. Diagn Pathol. 2014; 9:200.

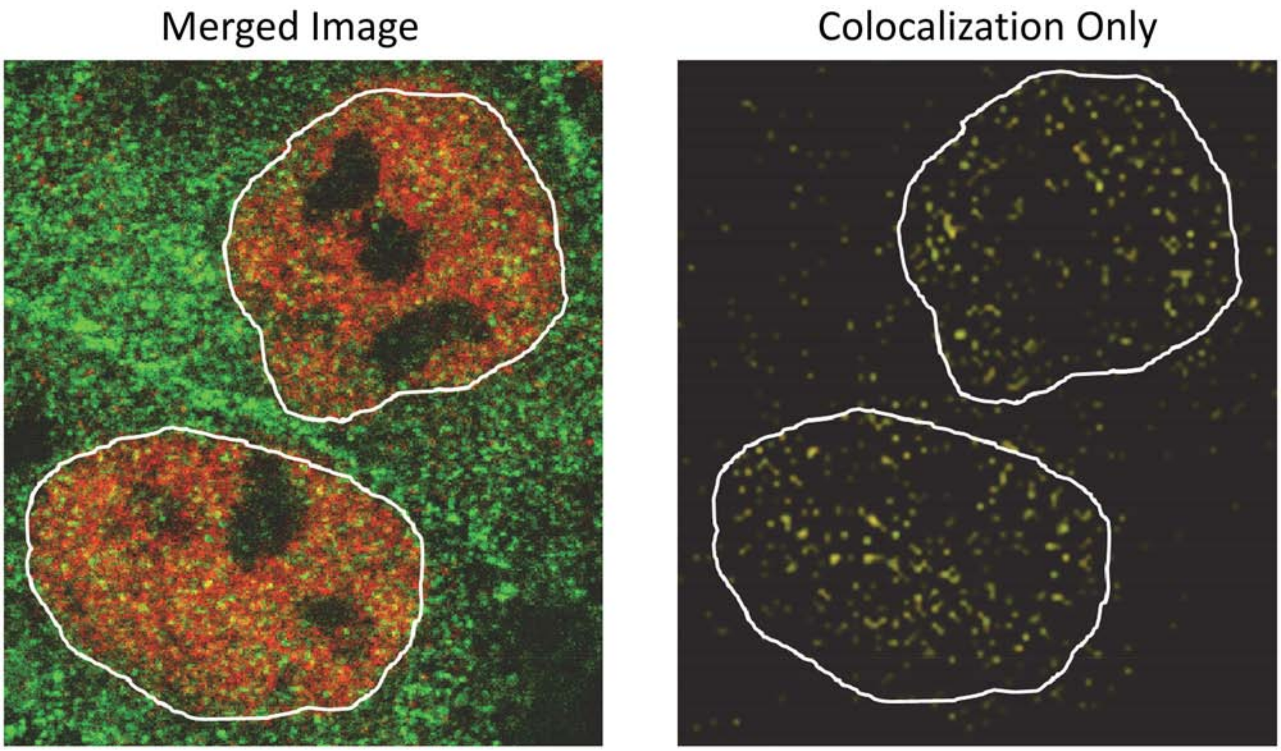

Supplementary Figure S1: Colocalization analysis between Src and p300 in Panc-1 cells. Src (green channel) and p300 (red channel) merged images from Figure 1 were expanded and an outline was drawn around the cell nuclei (left panel). Colocalization analysis (right panel, yellow) was performed using the Leica LAS AF software suite with the threshold set to 80% and the background set to 30% for both channels. An identical copy of the nuclear outlines was overlaid to denote the location of the nuclei in the right panel and demonstrate colocalization within the nucleus in each cell.

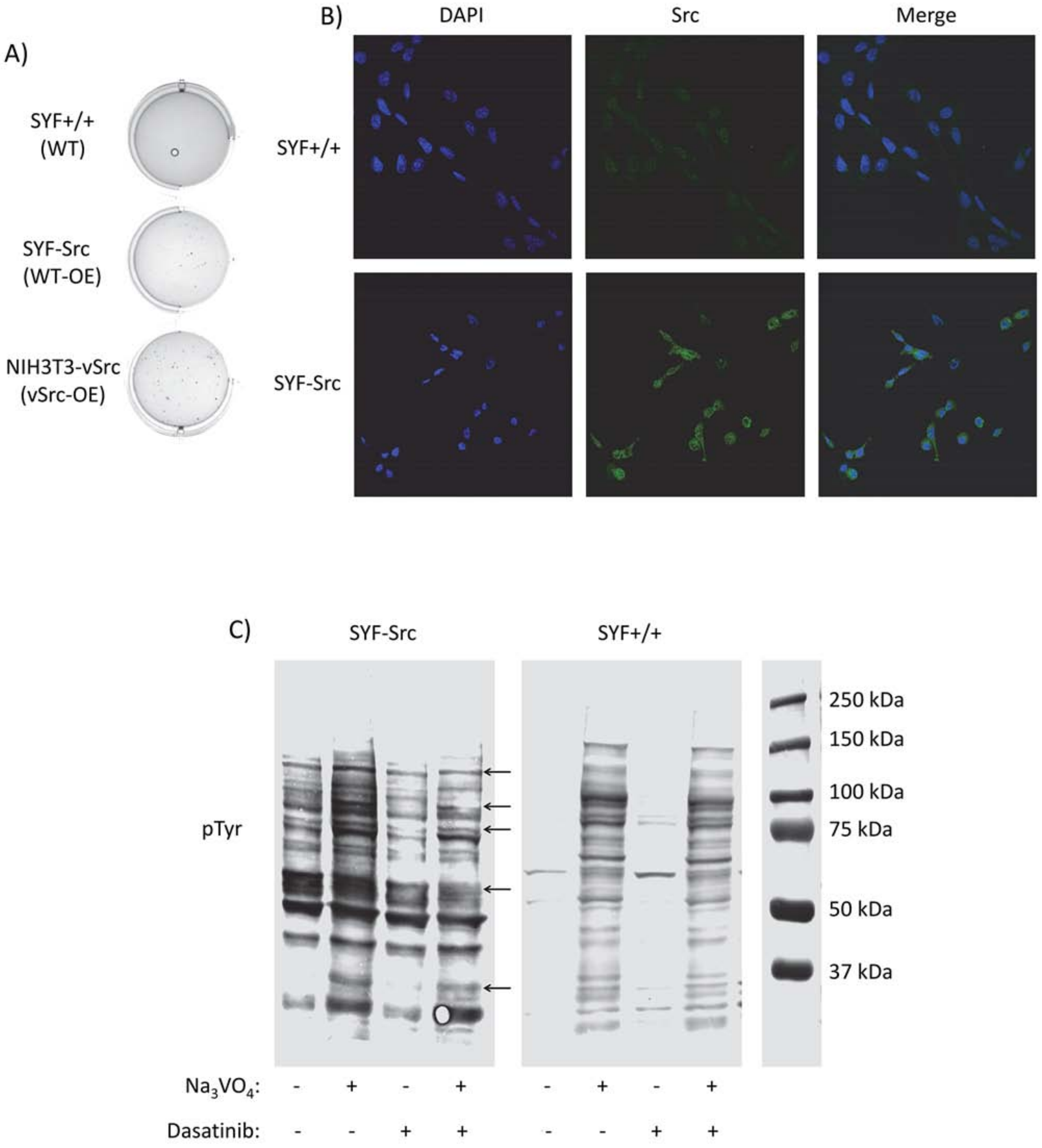

Supplementary Figure S2: Src localization and activity in SYF cell lines. A. Equal numbers of SYF+/+, SYF-Src, and NIH3T3vSrc cells were subjected to soft agar colony formation assay. Colonies were stained with Crystal Violet and imaged using the ProteinSimple FluorChem M. **B.** SYF+/+ and SYF-Src cells were seeded onto coverslips and stained simultaneously with Src antibody. Confocal images were acquired on a Leica TCS-SP5 microscope using identical settings for each image. Images are representative of > 5 fields in two independent experiments. **C.** Phosphotyrosine (pTyr) immunoblots of nuclei preparations from SYF-Src and SYF+/+ MEFs. Nuclei were harvested and subjected to *in vitro* tyrosine kinase activity assay in the presence or absence of 100 nmol/L dasatinib as indicated. Equal protein for each sample was analyzed by immunoblotting for general phosphotyrosine. Positions of differentially induced phosphoproteins are shown with arrows. Control lanes (-) represent nuclei incubated in 0.01% DMSO. Results are representative of two independent experiments.

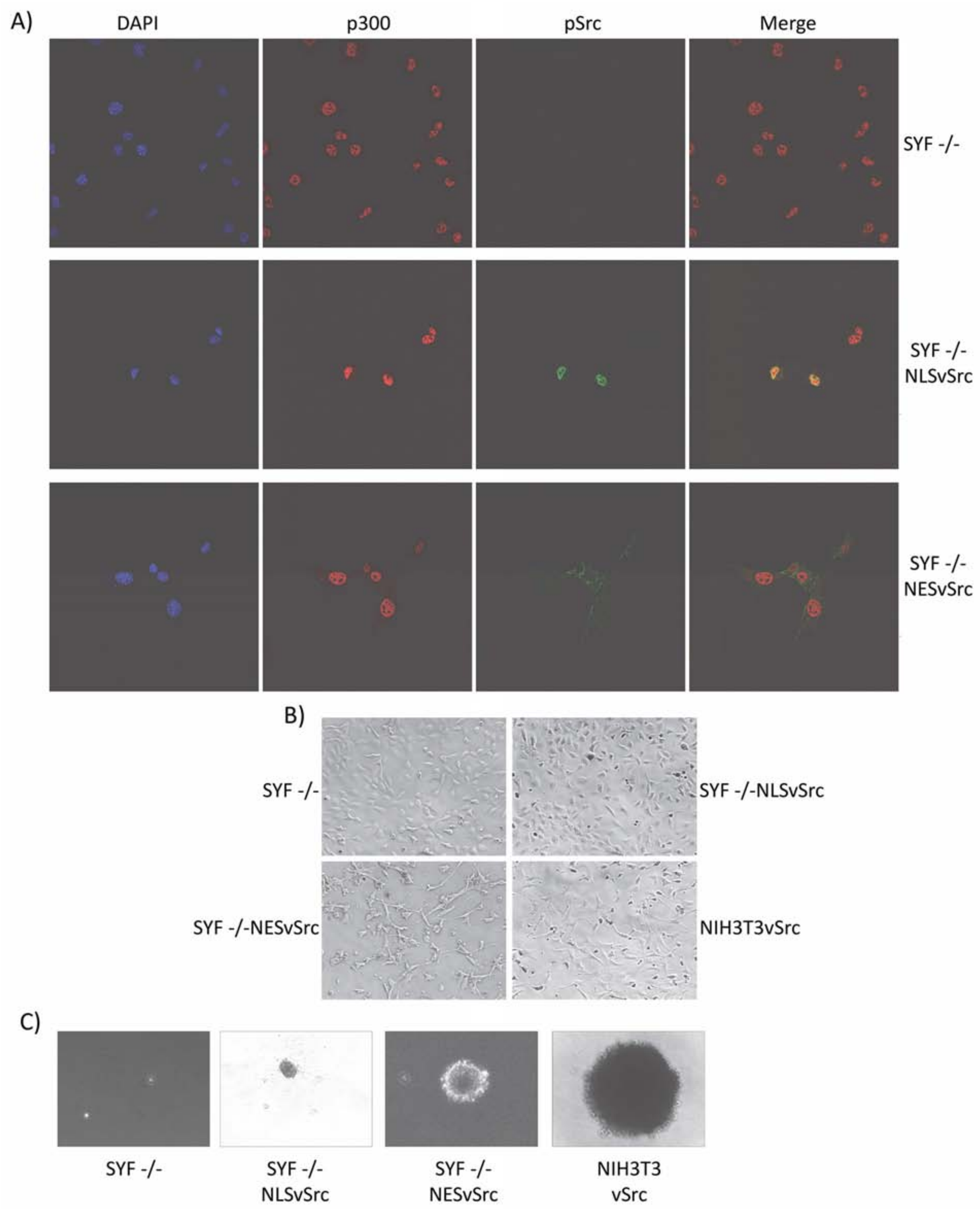

Supplementary Figure S3: Immunofluorescence imaging and morphology of cells stably transfected with vSrc constructs. A. SYF-/- MEFs or counterparts stable transfected with NLSvSrc or NESvSrc were seeded onto coverslips and subjected to simultaneous immunofluorescent staining against pSrc and p300. Images were acquired using identical settings on the Leica TCS-SP5. **B.** Gross morphology of SYF-/-, NLSvSrc, NESvSrc, and NIH3T3vSrc cells under 10X phase contrast microscopy. **C.** Representative images of soft agar colonies of SYF-/-, NLSvSrc, NESvSrc, and NIH3T3vSrc cells. Images were taken using a Zeiss Axiovert 200 microscope at 10X magnification. Data are representative of > 5 fields in two independent experiments.

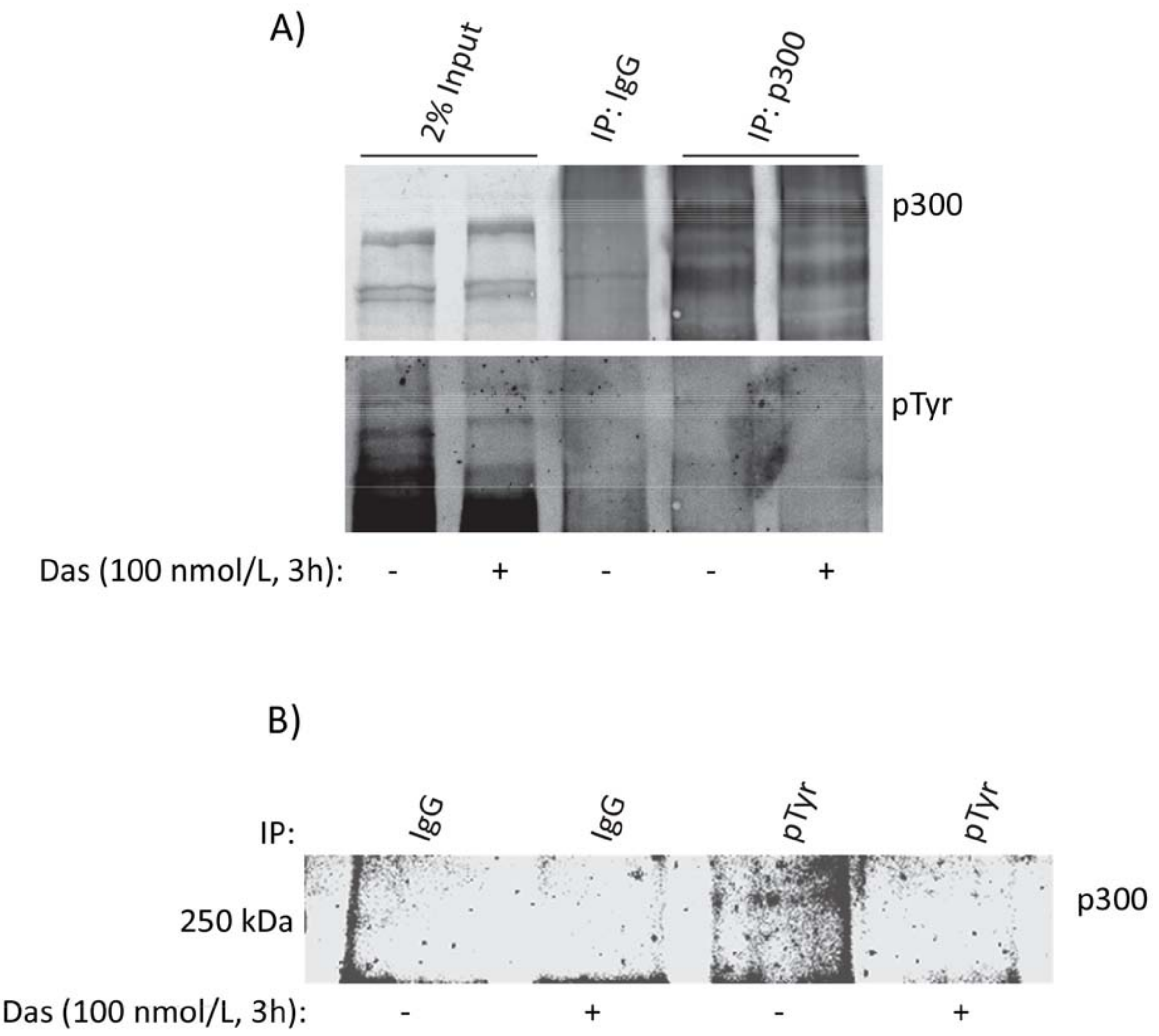

Supplementary Figure S4: Analysis of tyrosine phosphorylation of p300 in PDAC cells. A. Src-dependent tyrosine phosphorylation of p300 in the cell nucleus. Panc-1 cells were treated with 100 nmol/L dasatinib or untreated (DMSO) as indicated and nuclear lysates were prepared and subjected to immunoprecipitation for p300 and immunoblotting for p300 and phosphotyrosine (pTyr). 2% input lanes demonstrate equal loading of protein and equal p300 bands demonstrate equivalent immunoprecipitation efficiency between samples. Results are representative of two independent experiments. **B.** Panc-1 cells were treated with 100 nmol/L dasatinib or untreated (DMSO) and nuclear samples subjected to immunoprecipitation using general phosphotyrosine antibodies or IgG control and probed for p300. Positions of indicated proteins in gel are labeled. Control (-) lanes represent cells treated with 0.01% DMSO. Data are representative of three independent experiments.

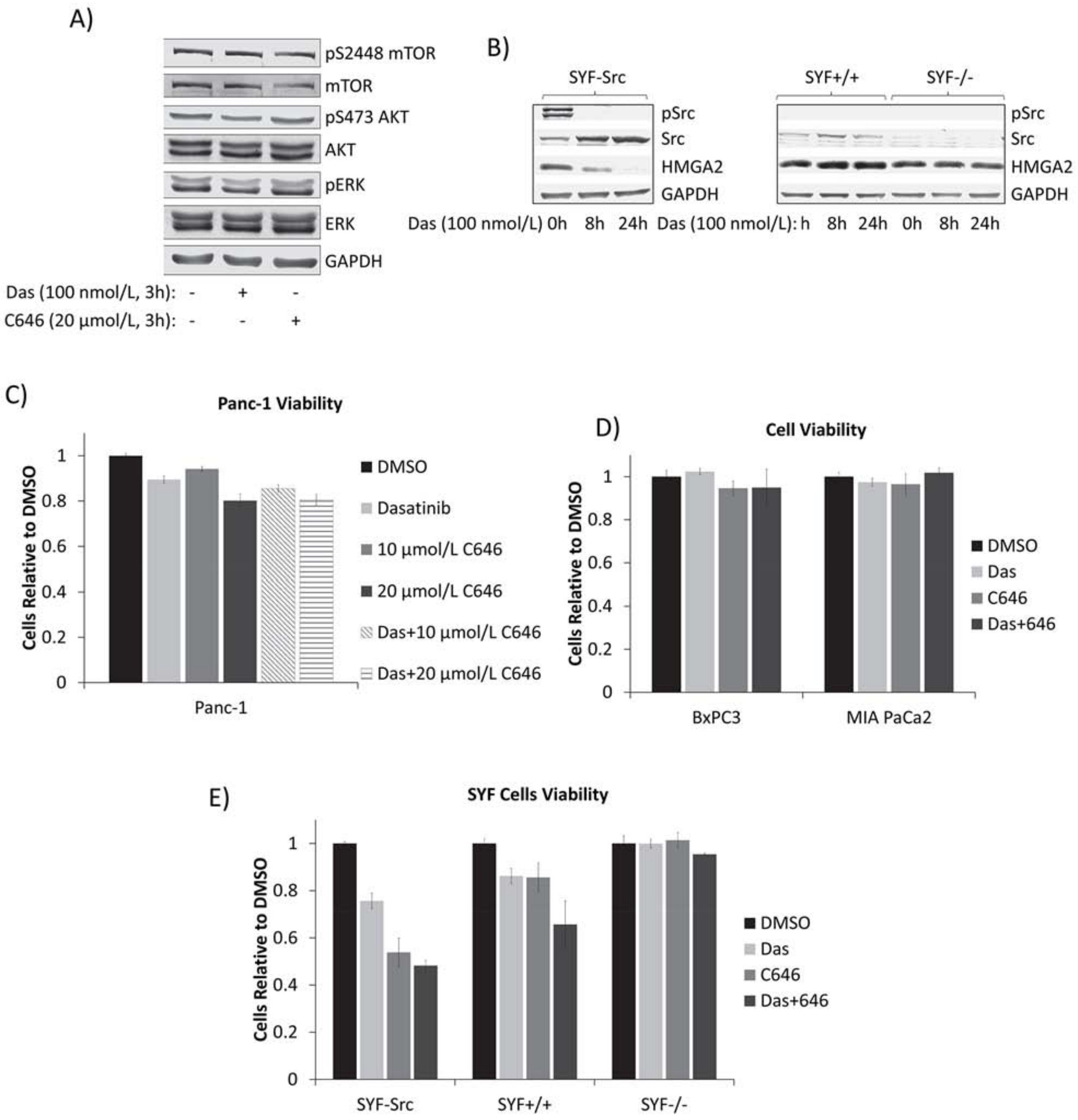

Supplementary Figure S5: Effects of Src or p300 inhibition on cell viability and on mTOR, AKT, and ERK signaling. A. Panc-1 cells were treated with the indicated concentrations of dasatinib or C646 for 3 hours and whole cell lysates were prepared and subjected to immunoblotting analysis for pS2448mTOR, mTOR, pS473AKT, AKT, pERK1/2, ERK1/2, and GAPDH. Results are representative of two independent experiments. **B.** SYF-Src, SYF+/+, and SYF-/- cells were treated for 8 or 24 hours with dasatinib and whole cells lysates were prepared and subjected to immunoblotting analysis for pSrc, Src, HMGA2, or GAPDH. Results are representative of three independent experiments. Positions of indicated proteins in gel are labeled. Control (-) lanes represent cells treated with 0.04% DMSO. **C.** Panc-1, **D.** BxPC3 and MIA PaCa-2, or **E.** SYF-Src, SYF+/+, and SYF-/- cells were seeded in 96-well plates in triplicate and treated with the indicated concentrations of dasatinib and C646 for 72 hours (C and D) or 48 hours (E). Relative cell number was then measured by CyQuant cell proliferation assay and represented as relative cell number compared to DMSO control. In all cases, initial cell density was determined by the cell number which would produce < 90% confluence in the control by 72 hours. Results are shown as mean ±SEM of three independent experiments. Control lanes (-, 0) represent cells treated with 0.05% DMSO.

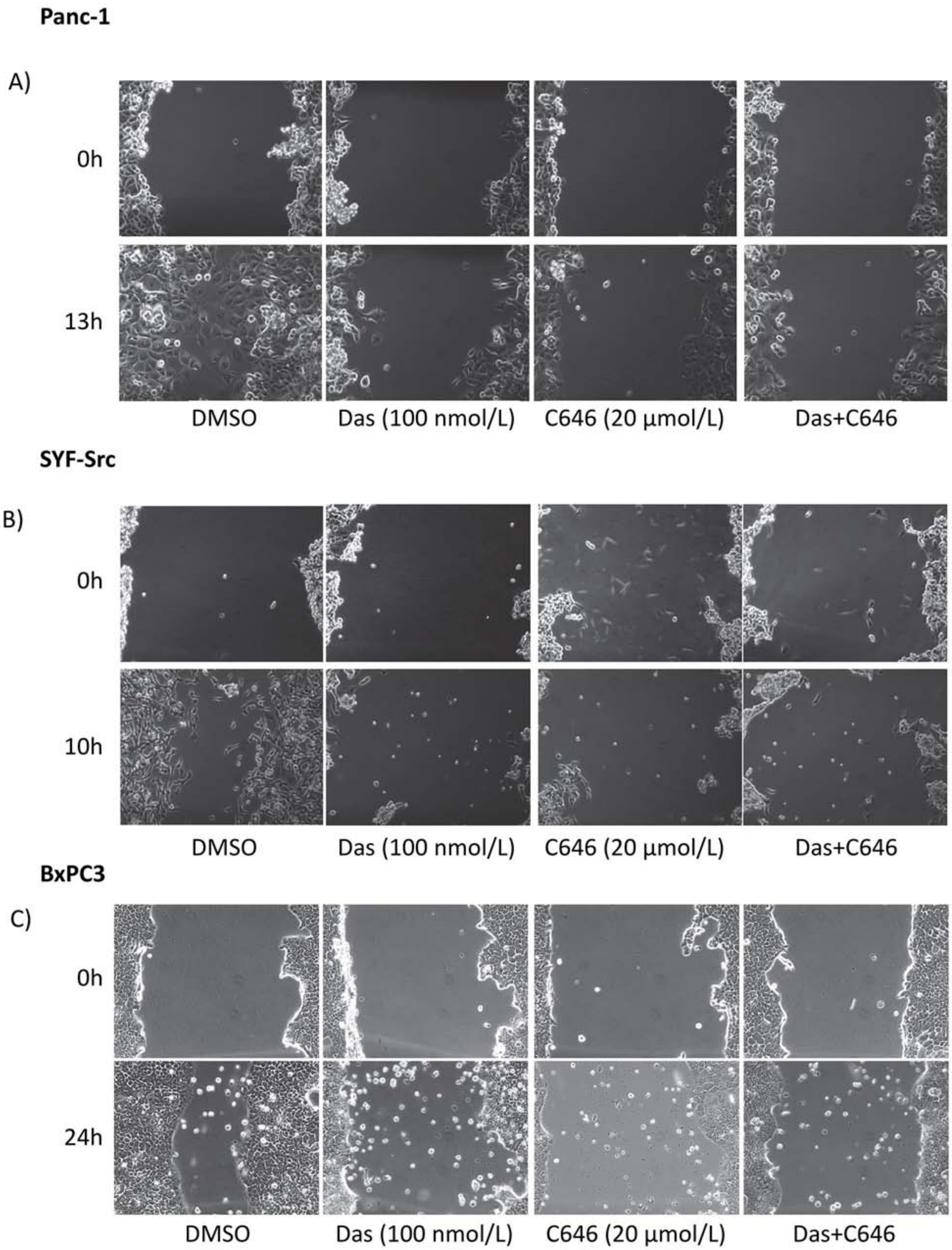
Panc-1
A)
0h
13h
DMSO
Das (100 nmol/L)
C646 (20 μmol/L)
Das+C646
SYF-Src
B)
0h
10h
DMSO
Das (100 nmol/L)
C646 (20 μmol/L)
Das+C646
BxPC3
C)
0h
24h
DMSO
Das (100 nmol/L)
C646 (20 μmol/L)
Das+C646

(*Continued*)

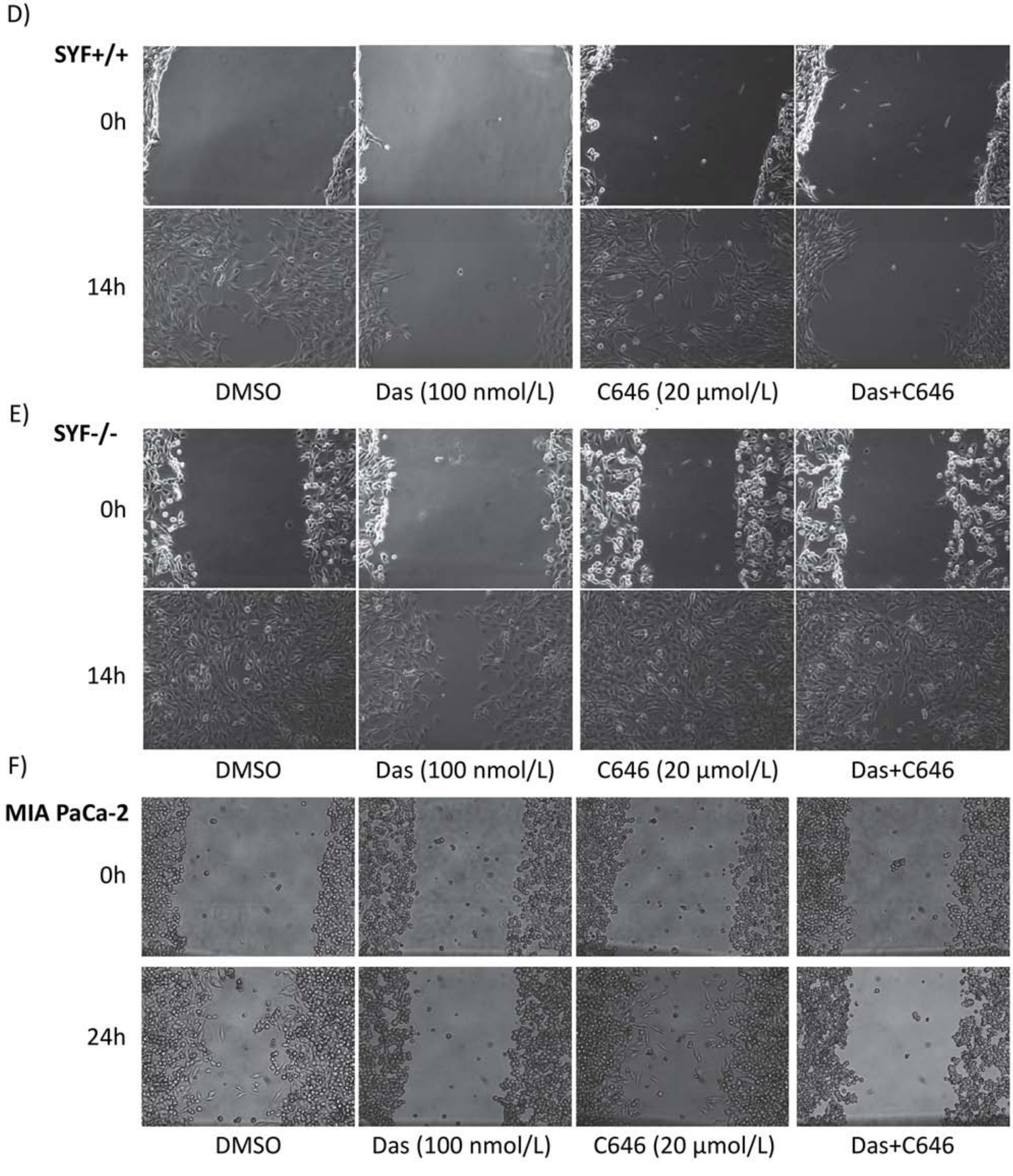

Supplementary Figure S6: Scratch assay images for effects of Src or p300 inhibitors. **A.** Panc-1, **B.** SYF-Src, **C.** BxPC3, **D.** SYF+/+, **E.** SYF-/-, or **F.** MIA PaCa-2 cells were seeded as confluent monolayers, and then wounded and treated with 100 nmol/L dasatinib or 20 µmol/L C646 for 10–24 hours as indicated. Wounded areas were imaged in phase contrast with the Zeiss Axiovert 200 microscope at 10X. Images are representative of three independent experiments.

Supplementary Table S1: Peptides detected from immunoprecipitation (IP)-mass spectrometry. Specific peptides detected in the Src IP but not in the IgG control were expressed along with the total peptides detected. Protein name and brief function are also represented.

Specific Peptides	Total Peptides	Protein Name	Function
8	17	USO1	Vesicular Trafficking
5	5	p300	Coactivator and histone acetyltransferase
4	9	GSTP1	Regulator of redox
3	5	HSPA1L	Chaperone protein
3	4	ARHGEF7	GEF for Rac1
2	18	ALY/REF	mRNA export factor
